# Status and associated characteristics of HIV disclosure among people living with HIV/AIDS in Liangshan, China: A cross-sectional study

**DOI:** 10.1097/MD.0000000000016681

**Published:** 2019-08-02

**Authors:** Yao Yin, Hui Yang, Xia Xie, Huan Wang, Anliu Nie, Hong Chen

**Affiliations:** West China School of Nursing and Department of Nursing, West China Hospital, Sichuan University, Chengdu, Sichuan, China.

**Keywords:** China, disclosure, human immunodeficiency virus (HIV), people living with HIV/acquired immune deficiency syndrome

## Abstract

Human immunodeficiency virus (HIV) disclosure is a prerequisite to get access to antiretroviral therapy (ART) and social support. Increased disclosure of HIV status has been shown to reduce mother-to-child transmission and high-risk sexual behaviors. Limited studies were conducted to get an insight into HIV disclosure among people living with HIV/acquired immune deficiency syndrome (AIDS) (PLWHA) in Liangshan.

Our study aimed to investigate the status and associated characteristics of HIV disclosure among PLWHA in Liangshan.

We conducted a cross-sectional study using a stratified, convenience sampling method from August to December in 2017. All of the participants were from Liangshan, a typical impoverished mountainous area which also has a long history of drug production and drug trade. Each participant completed a structured questionnaire including HIV disclosure status, demographic and HIV-related characteristics, social support, and perceived HIV-related stigma. We performed a binary regression analysis to detect associated characteristics of HIV disclosure among PLWHA in Liangshan.

A final sample size of 318 participants was included in this study. The overall prevalence of HIV disclosure was 83.6% (266/318). In binary logistic regression analysis, PLWHA who had higher educational levels, and got infected by sexual transmission were less likely to disclose their HIV status (both *P* < .05). HIV nondisclosure was correlated with a higher level of perceived HIV-related stigma (*P* < .01).

The prevalence of HIV disclosure was relatively low in Liangshan. Healthcare workers are suggested to conduct more counseling and education to promote safe sexual behaviors and reduce perceived stigma among PLWHA, then enhance HIV serostatus disclosure.

## 1 Introduction

Nationally, the human immunodeficiency virus (HIV)/acquired immune deficiency syndrome (AIDS) epidemic in China is categorized as a low prevalence (0.037%).^[1]^ However, HIV new infections in China account for the second proportion of HIV new infections in Asia and the Pacific.^[2]^ In China, the HIV/AIDS epidemic is unevenly distributed across regions and provinces. The northwest and southwest China are the most HIV-affected regions.^[3]^ Liangshan is an autonomous prefecture of Sichuan Province, which is located in southwest China. Seventeen counties and cities comprise the Liangshan Yi prefecture, with 5 counties having an HIV high prevalence (>1%).^[4]^ It was reported that 29,778 people living with HIV/AIDS (PLWHA) lived in Liangshan, accounted for 38% of all PLWHA in Sichuan Province in 2015. Particularly, long-term poverty and a long history of drug production and drug trade were linked with high HIV prevalence in this area.^[4–6]^

The HIV disclosure is reported to be one of the effective ways for HIV prevention and control. Evidence indicated that safer sexual behavior and less mother-to-child transmission resulted from HIV disclosure.^[7,8]^ HIV disclosure was associated with early initiation and better adherence of antiretroviral therapy (ART), freedom to use ART drugs, care continuity, ultimately can lead to better outcomes of CD4^+^ cell counts, and less failure of treatment.^[8–12]^ Further, HIV disclosure was correlated to reduced mortality of HIV-infected individuals on ART.^[13]^ PLWHA could get emotional, moral, and financial support after disclosure of HIV serostatus.^[12,14,15]^

However, HIV disclosure could also bring some negative outcomes. It was possible for PLWHA to experience disrupted relationships with families and communities, isolation, criticism, and ostracism by family members, abuse, violence, divorce, or separation from partners, rejection by friends, stigma and discrimination by healthcare workers and public, losing their jobs, and being out as PLWHA after HIV disclosure.^[8,9,12,14,16–18]^ Nevertheless, negative reactions were relatively low, and 76.1% of PLWHA declared that they did not regret to disclose their HIV serostatus.^[9,19]^

Research indicated that HIV disclosure was different by regions and countries.^[8]^ Numerous research explored associated characteristics with HIV disclosure. Individual characteristics including gender, age, marital status, occupation, educational level, and religion played a mixed role in HIV disclosure.^[20–26]^ Living alone, belonging to minority groups, and having casual partners were associated with HIV nondisclosure.^[26–28]^ Although studies suggested that disclosure was a gradual process, the findings of the correlation between time since 1st HIV diagnosis and HIV disclosure were mixed.^[29–31]^ Besides, PLWHA who did not receive ART, had lower CD4^+^ cell counts, had a shorter duration of treatment, and get infected by sexual behaviors were less likely to disclose their HIV status to others.^[32–34]^ Psychologic characteristics including higher levels of perceived HIV-related stigma and severe depression status were associated with HIV nondisclosure.^[8,35]^ Social context including a higher level of social support, more close friends, and living within the monogamous family were related to HIV disclosure.^[29,32,34]^ Moreover, HIV serostatus disclosure among PLWHA was related to advising and counseling from healthcare workers.^[12,36]^

Limited studies were conducted to get an insight into HIV disclosure among PLWHA in Liangshan. Therefore, our study aimed to investigate the status and associated characteristics of HIV disclosure among PLWHA.

## 2 Methods

### 2.1 Ethics statement

West China Hospital Medical Ethics Committee approved the protocol and consent procedure of the study (No. 430[2017]). We received the written informed consent from all participants.

### 2.2 Study population

We conducted a cross-sectional study by a 2-stage sampling method from August to December in 2017. Firstly, Liangshan was divided into HIV high -prevalence areas (5 counties) and low prevalence areas (12 counties and cities) according to the HIV/AIDS epidemic. The HIV high-prevalence areas were defined as the areas with HIV prevalence that exceeded 1%. We randomly selected Xichang city and Zhaojue county as the presentative site of HIV high-prevalence areas and HIV low prevalence areas, respectively. Secondly, we recruited participants by convenience sampling from the Center for Disease Control and AIDS management centers. According to the inclusion criteria, participants had to meet diagnostic criteria for HIV/AIDS released by the Chinese Ministry of Health in 2008.^[37]^ Participants also should be 18 and older, and consent to participate. Participants with mental disorder or mental deficiency were excluded.

### 2.3 Data collection

Investigators were trained about the uniform instruction, purpose, contents, the potential risks, and benefits of the study. Prior to implementation, the investigators informed all participants of the study purpose, contents, potential risks, and benefits by using uniform instruction. Participants filled the anonymous questionnaires. Face-to-face structured interviews were conducted if the participants were illiterate. All questionnaires were immediately checked after completion.

### 2.4 Demographic and HIV-related characteristics

Demographic characteristics included gender, age, ethnicity, marital status, residence, employment status, and household income monthly per capita (HIMPC). HIV-related characteristics included HIV prevalence area, disclosure status, ART status, route of HIV infection, time since HIV diagnosis, and clinical symptoms.

### 2.5 Chinese version of Medical Outcomes Study Social Support Survey (MOS-SSS-C)

Sherbourne and Stewart developed the scale for evaluating the social support of patients with chronic diseases.^[38]^ Based on Doris and colleagues’ work,^[39]^ simplified Chinese version of MOS-SSS-C was adapted and applied to HIV-infected patients by Li.^[40]^ It was reported good reliability (*α* = 0.889) and construct validity.^[40]^ This 19-item scale measures 4 domains of social support, including tangible support (4 items), affectionate support (3 items), positive social interaction (3 items), and emotional and informational support (8 items). A higher total score indicates more social support.

### 2.6 Berger HIV stigma scale

We used the HIV stigma scale to measure the stigma perceived by PLWHA. It was developed by Berger and adapted and validated in China.^[41,42]^ The Chinese version of the HIV stigma scale was reported adequate content validity and construct validity. Its internal consistency reliability was also supported (*α* = 0.945).^[42]^ This 40-item instrument consists of four subscales: personalized stigma, disclosure concerns, negative self-image, and concern with public attitudes. Each item can be responded by a 4-point Likert scale (strongly disagree, disagree, agree, strongly agree). Two items are reverse scored (e.g., Having HIV makes me feel I’m a bad person), and a higher total score means higher levels of stigma perceived by PLWHA.

### 2.7 Statistical analysis

All data analyses were performed using SPSS software (version 22.0; IBM Corp, Armonk, NY). The demographic and HIV-related characteristics were described by frequency and percentage, median, and interquartile range (IQR). We conducted the Mann–Whitney *U* test or Chi-squared test to explore the correlation between basic characteristics and HIV disclosure. We conducted a binary logistic regression analysis to determine the associated characteristics of HIV disclosure. A *P* < .05 was set as statistically significant.

## 3 Results

### 3.1 Demographic and HIV-related characteristics

Table 1 presents the demographic and HIV-related characteristics of 318 PLWHA included in final analyses. The median age of them was 37 years old (range: 19–75). Most participants (n = 195, 61.3%) were males; 76.7% (n = 244) were Yi ethnicity; more than half of participants (n = 167, 52.5%) were illiterate; 66.7% (n = 212) were married; 83.3% (n = 265) lived in rural areas; 91.2% (n = 290) were employed; only 21.7% of PLWHA had HIMPC more than 500 yuan (1$ = 6.9 yuan). The majority of participants (83.6%, n = 266) disclosed their HIV status to others; 83.6% (n = 266) received ART; 72.6% (n = 231) had clinical symptoms. Sexual transmission was the main route of HIV transmission, accounting for 46.2% (n = 147) of the participants. The median disease duration was 54 months (range: 2 months to 21 years). The median score of MOS-SSS-C and perceived HIV-related stigma was 55 and 110, respectively.

**Table 1:**
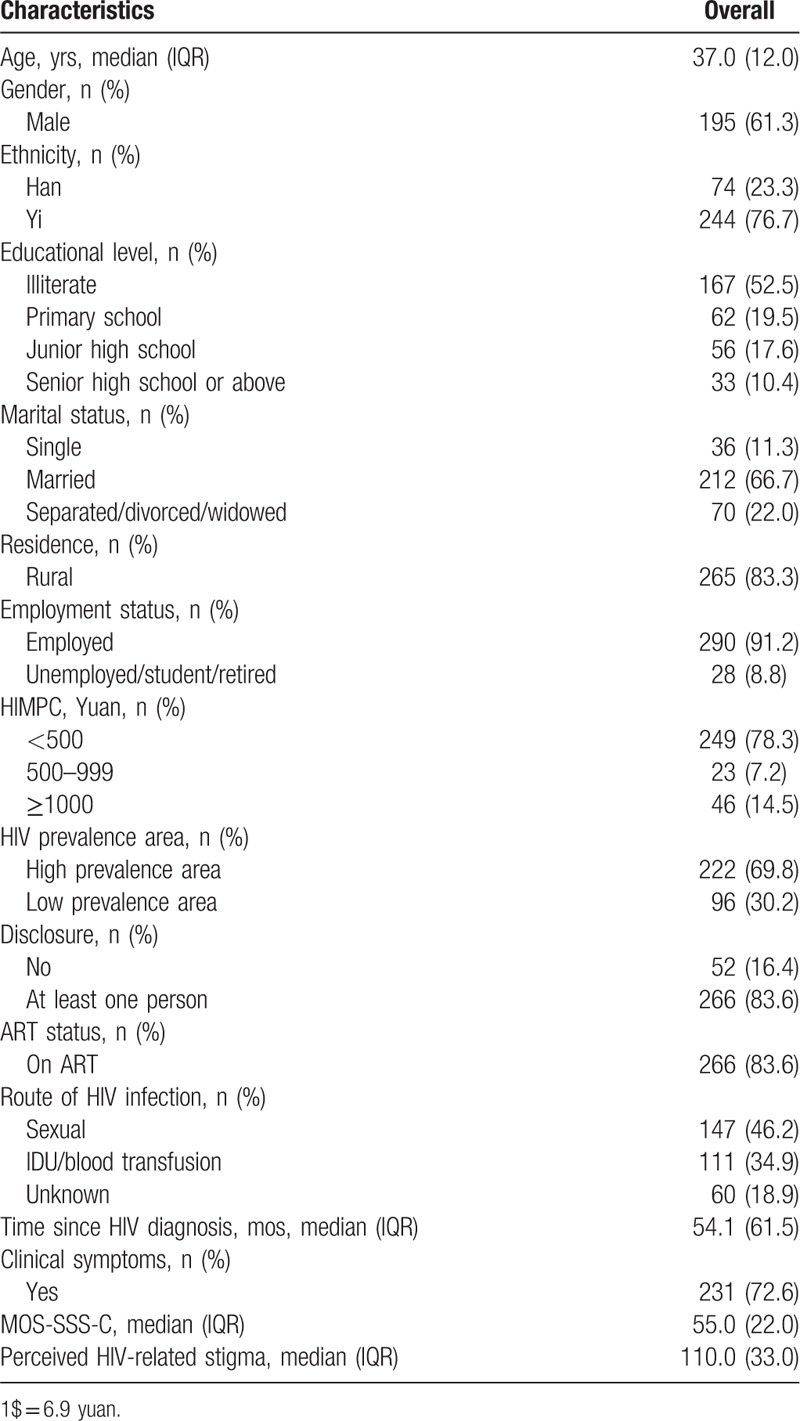
Sample demographic and HIV-related characteristics.

### 3.2 Correlation between basic characteristics and HIV disclosure

The potential factors correlated to HIV disclosure are shown in Table 2. PLWHA who were members of the Yi ethnicity group, had a lower educational level (illiterate vs primary school or junior high school or senior high school or above), were rural residents, were employed, and were poorer (HIMPC < 500 yuan vs HIMPC ≥ 500 yuan) were more likely to disclose their HIV status. Additionally, participants who were living in HIV high-prevalence areas, did not receive ART, got infected by injection drug use (IDU)/blood transfusion (IDU/blood transfusion vs sexual behavior or unknown), had clinical symptoms were more likely to disclose their serostatus to others. Older PLWHA and those who had higher levels of perceived HIV-related stigma were significantly associated with HIV nondisclosure.

**Table 2:**
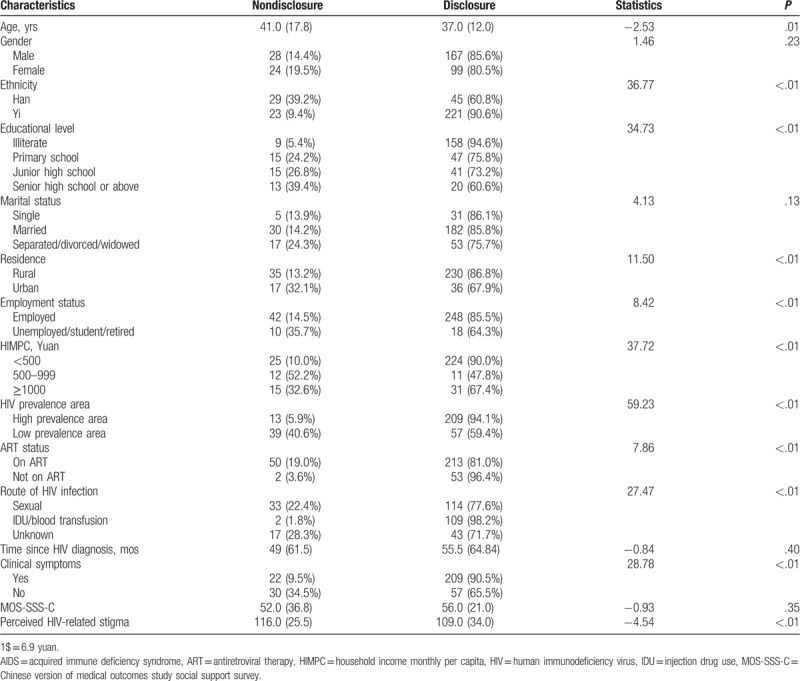
Correlation between basic characteristics and HIV disclosure of people living with HIV/AIDS.

### 3.3 Binary logistic regression of HIV disclosure

As shown in Table 3, the participants who had higher educational levels and had higher levels of perceived HIV-related stigma were less likely to disclose their HIV status to others. Compared with participants who got infected by sexual behaviors, PLWHA who got infected by IDU/blood transmission were more likely to disclose.

**Table 3:**
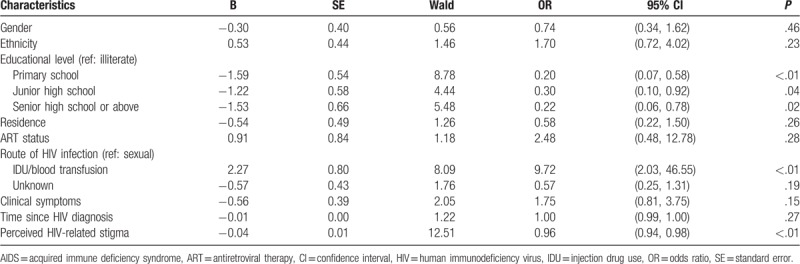
Binary logistic regression of HIV disclosure of people living with HIV/AIDS.

## 4 Discussion

Overall, 83.6% of the participants disclosed their HIV status to at least one person in Liangshan. It was lower than the findings (97–100%) in other provinces that have similar HIV epidemic as Liangshan.^[30,43]^ Lower disclosure in Liangshan could lead to a higher risk of HIV transmission and discontinuity treatment, subsequently lead to negative health outcomes and harder control of HIV prevention and control.^[12,44,45]^

Although many studies reported that gender played an important role in HIV disclosure,^[22,46,47]^ we did not find consistent results in this study. Notably, some researchers reported that female participants were less likely to disclose to sexual partners than male participants.^[24,48]^ Female positives were reported to get less acceptance, care, and support by their spouses.^[49]^ Gender-based violence could also be an explanation of this phenomenon.^[50]^ Moreover, females tended to disclose to the family member first, but males tended to disclose to spouses/partners first.^[25]^ Further studies could focus on the gender difference in the motivation, the process, and to whom of the disclosure.

Compared with illiterate participants, PLWHA who had higher educational levels were less likely to disclose to others about their HIV status. A possible explanation might be that PLWHA who were more educated had increased self-stigma, which cause fear of disclosure.^[51]^ Also, 99.4% and 95.8% of the illiterate participants lived in rural areas and had an HIMPC <500 yuan in this study. In China, national policy, “Four Free and One Care,” documented that PLWHA living in rural areas or impoverished individuals without insurance residing in urban areas can receive free antiretroviral drugs treatment and financial assistance from the government.^[52]^ Only based on disclosure could PLWHA get this material and financial support. A retrospective cohort reported that PLWHA who attained higher educational levels had a higher risk of loss to follow-up when receiving ART.^[53]^ Therefore, higher educated PLWHA need intervention to improve their disclosure and retain in ART.

Compared with participants who got infected by sexual behaviors, participants who got infected by IDU/blood transfusion were more likely to disclose their HIV status. It can be explained that Liangshan has a long history of drug production and drug trade.^[54]^ And PLWHA who got infected by sexual behaviors feared to be linked to promiscuity in sexual and moral judgment after HIV disclosure. Researchers also applied the Asian Epidemic Model (AEM) to predicted that sexual transmission would be the major route of new infections in Liangshan.^[55]^ Therefore, healthcare workers should conduct counseling and education to promote safe sexual behavior and HIV disclosure among PLWHA.

We found that PLWHA with a higher level of perceived stigma were less likely to reveal their HIV serostatus to others. It was consistent with previous studies suggesting that HIV nondisclosure was associated with fear of stigma and discrimination and social exclusion.^[56,57]^ A meta-analysis also showed a negative and stable correlation between stigma and disclosure.^[58]^ Conversely, a systematic review indicated that the effect of stigma on HIV disclosure varied according to types of stigma, measurements of stigma, and personal background and health.^[59]^ To address the mentioned problems, more homogeneous studies should be conducted to develop stigma-reduction interventions, then to promote HIV disclosure.

Limitations were inevitable in our study. We only explored the prevalence of HIV disclosure among PLWHA. Further studies should focus on the differences among whom PLWHA disclosed to and types of selective disclosure. This cross-sectional study could only reflect the correlation rather than the causation. Longitudinal studies need to be conducted. As the subjects were recruited by a stratified, convenience sampling method, the universality of study results should be cautious. Reporting bias should also be considered because of relying on self-reported disclosure status in this study.

## 5 Conclusion

We found that the prevalence of HIV disclosure was relatively low in Liangshan. The study revealed that PLWHA who had higher educational levels, got infected by sexual transmission, and had higher levels of perceived HIV-related stigma were less likely to disclose their HIV status. And there was no significant relationship between gender and HIV disclosure. Interventions focusing on safe sexual behaviors and reducing perceived HIV-related stigma are needed in Liangshan.

## Acknowledgment

The authors express sincere gratitude to patients who participated in this research and investigators who helped data collection.

## Author contributions

**Conceptualization:** Yao Yin, Hui Yang, Xia Xie, Huan Wang, Anliu Nie, Hong Chen.

**Data curation:** Yao Yin, Hui Yang, Xia Xie, Huan Wang, Anliu Nie, Hong Chen.

**Formal analysis:** Yao Yin.

**Investigation:** Yao Yin, Hui Yang, Xia Xie, Huan Wang, Anliu Nie, Hong Chen.

**Methodology:** Yao Yin, Hui Yang, Xia Xie, Huan Wang, Anliu Nie, Hong Chen.

**Project administration:** Hong Chen.

**Supervision:** Hong Chen.

**Writing – original draft:** Yao Yin.

**Writing – review & editing:** Yao Yin, Hui Yang, Xia Xie, Huan Wang, Anliu Nie, Hong Chen.
